# Pb-Induced Eryptosis May Provoke Thrombosis Prior to Hemolysis

**DOI:** 10.3390/ijms23137008

**Published:** 2022-06-23

**Authors:** Qiushuo Jin, Chunyang Yao, Yiying Bian, Jingbo Pi

**Affiliations:** School of Public Health, China Medical University, No. 77 Puhe Road, Shenyang North New Area, Shenyang 110122, China; jinqiushuo1998@126.com (Q.J.); yaochunyang1995@163.com (C.Y.)

**Keywords:** lead (Pb), ROS-independent, intracellular calcium level, eryptosis, shape changes, venous thrombosis mice model

## Abstract

Lead (Pb) is a common metal, which can be toxic to the human body via the pollution of water or food, and can cause anemia and other diseases. However, what happens before hemolysis and anemia caused by Pb poisoning is unclear. Here, we demonstrated Pb can cause procoagulant activity of erythroid cells leading to thrombosis before hemolysis. In freshly isolated human erythroid cells, we observed that Pb resulted in hemolysis in both concentration- and time-dependent manners, but that no lysis occurred in Pb-exposed erythroid cells (≤20 μM for 1 h). Pb treatment did not cause shape changes at up to 0.5 h incubation but at 1 h incubation echinocyte and echino-spherocyte shape changes were observed, indicating that Pb can exaggerate a concentration- and time-dependent trend of shape changes in erythroid cells. After Pb treatment, ROS-independent eryptosis was shown with no increase of reactive oxygen species (ROS), but with an increase of [Ca^2+^]_i_ and caspase 3 activity. With a thrombosis mouse model, we observed increased thrombus by Pb treatment (0 or 25 mg/kg). In brief, prior to hemolysis, we demonstrated Pb can cause ROS-independent but [Ca^2+^]_i_-dependent eryptosis, which might provoke thrombosis.

## 1. Introduction

Lead (Pb) is one of the most abundant heavy metals on earth and a highly toxic pollutant. The sources of lead exposure in China include e-waste, drinking water, traditional drugs, and industrial emissions [1]. Pb exposure could affect multiple organs in the body including the kidneys, liver, and central nervous system. In addition, there is a lot of evidence that Pb exposure is associated with anemia [2] and cardiovascular disease [3,4]. Anemia is the most common issue caused by Pb exposure, and therefore, people often pay less attention to some more important health problems which might occur prior to anemia, such as thrombosis.

Since the early 1970s, the Center for Disease Control (CDC) has supported relevant departments to develop lead poisoning prevention programs. With the implementation of the lead poisoning prevention plan, the safety level of blood lead is continuously reduced with the increasing new discoveries of multiple toxic effects. Since 2015, the safety range of children’s blood lead has been set at 5 μg/dL. If a laboratory blood lead test shows 10 μg/dL, children are determined to be at the “concern level” of blood lead content [5,6]. Therefore, it is not difficult to predict that the safety range of blood lead levels will decrease in the future with new discoveries of toxic effects other than anemia.

Production of reactive oxygen species (ROS), as a common factor related to Pb exposure, also correlates with anemia [7,8]. In an ROS-dependent manner, Pb exposure could lead to cell death and energy depletion [9]. Pb exposure also induces hemolysis, which is linked to an increase in oxidative damage leading to the production of ROS [10,11]. Moreover, ROS also participates in blood cell dysfunction and thrombosis [12,13].

Oxidative stress can trigger the depletion of antioxidant activity and promote the process of erythroid cell apoptosis [2,14,15,16]. In humans with Pb exposure, 95% of absorbed Pb is accumulated into erythroid cells, which are known to be a prime target for Pb toxicity [17]. Oxidative stress could induce an increment of the erythroid cells apoptosis, which is defined as eryptosis, and might be regulated through the rise of intracellular calcium concentration ([Ca^2+^]_i_) [18]. Eryptosis is characterized by cell shrinkage and exposure of phosphatidylserine (PtdSer) residues at the cell surface [19]. Moreover, the delivery of microvesicles (MVs) is considered as one part of eryptosis [20], and some studies have also established that MVs play an important role in hemostasis and thrombosis [21].

With this in mind, we hypothesized that ROS and intracellular calcium can regulate eryptosis which could promote the procoagulant activity of erythroid cells caused by Pb exposure prior to hemolysis. Here, we investigated the mechanisms underlying lead-induced thrombosis before hemolysis occurred. And with respect to eryptosis, shape changes of erythroid cells were accompanied by PtdSer exposure and MV generation in an ROS-independent but [Ca^2+^]_i_-dependent eryptosis with activated caspase 3. Furthermore, Pb exposure can induce thrombin generation, erythroid cells aggregation, and erythroid cells adhesion to endothelial cells. Indeed, in vivo relevancy was further confirmed by using a venous thrombosis mice model.

## 2. Results

### 2.1. Effects of Pb on Hemolysis in Freshly Isolated Human Erythroid Cells

**Figure 1:**
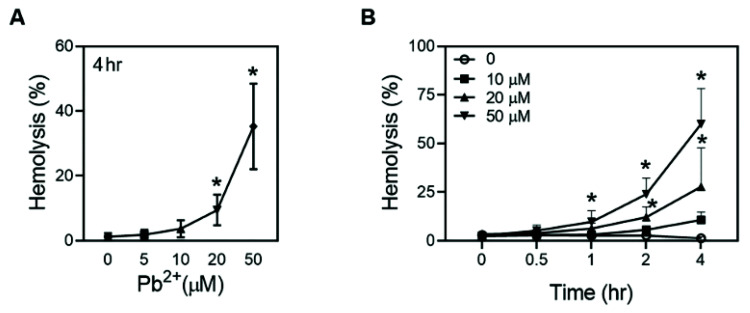
Effects of Pb on hemolysis in freshly isolated human erythroid cells. After erythroid cells were treated with distilled water (DW; control) or various concentrations (0, 5, 10, 20, and 50 μM) of Pb, hemolysis was evaluated at 540 nm. (**A**) Concentration-dependent Pb was treated for 4 h (*n* = 4). (**B**) Time- and concentration-dependent courses were measured with various concentrations of Pb up to 4 h (*n* = 4–7). Values are the mean ± SD of 4–7 experiments. * Statistical difference compared with control (*p* < 0.05).

First, we examined the hemolytic responses of human erythroid cells exposed to different concentrations of Pb. Treatment with Pb for 4 h induced hemolysis in a concentration- and time-dependent manner as compared to that with a vehicle (Figure 1A,B). As shown in Figure 1B, with the increase of exposure time, the minimal toxic level of Pb was being lower. Here, no hemolysis (≤10%) was observed after Pb treatment with concentrations ≤20 μM at 1 h.

### 2.2. Pb Treatment Augments Eryptosis and Shape Changes of Human Erythroid Cells

**Figure 2:**
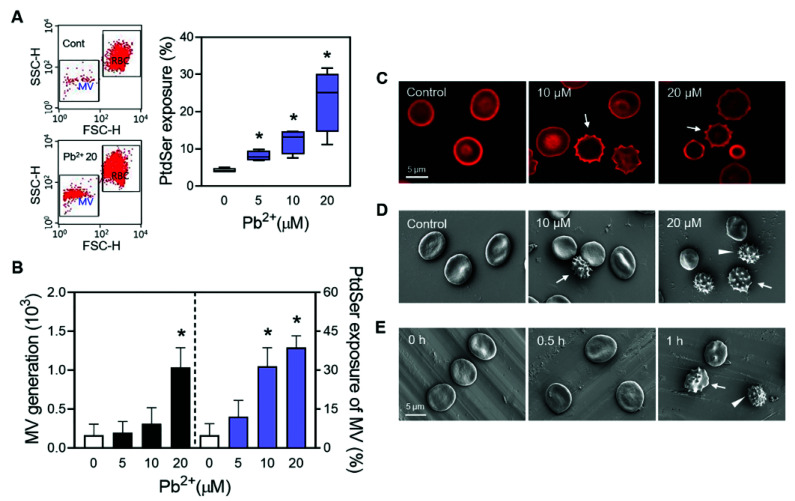
Pb treatment augments eryptosis and shape changes of human erythroid cells. After isolated human erythroid cells were treated with various concentrations (0, 5, 10, and 20 μM) of Pb. (**A**) PtdSer exposure (*n* = 4). (**B**) MV generation and PtdSer exposure of MV were concentration-dependent promoted, which was determined by using flow cytometry. (**C**–**E**) Erythroid cells shape changes were examined by using a confocal microscope (*n* = 4) and a scanning electron microscope (*n* = 3), respectively. Values are the mean ± SD of 3–4 experiments. Scale bars (**C**–**E**): 5 μm. * Statistical difference compared with control (*p* < 0.05).

Eryptosis provides the erythroid cells with another form of cell death other than hemolysis [22]. To explore the events prior to hemolysis, Pb treatment of no more than 20 μM for 1 h was conducted in the following experiments. With flow cytometric determination, both externalized PtdSer labeled with annexin V-FITC (Figure 2A) and generated MV from erythroid cells (Figure 2B, left) were increased in a concentration-dependent manner. In addition, PtdSer externalization of released MV also showed a concentration-dependent increase (Figure 2B right). Loss of phospholipid asymmetry is always known to be accompanied by morphological changes in erythroid cells [23], with discocytic shapes changing into echinocytic and spherocytic shapes in a concentration dependency caused by a stimuli [24,25], which matched well with our confocal observation and SEM observation (Figure 2C,D). Moreover, we observed Pb treatment could not cause shape changes at up to 0.5 h incubation, but at 1 h incubation echinocyte (arrows) and echino-spherocyte (arrow heads) changes were observed (Figure 2E), indicating that Pb can exaggerate a concentration- and time-dependent trend in shape changes of erythroid cells.

### 2.3. ROS-Independent but [Ca^2+^]_i_-Dependent Eryptosis by Pb-Treated Erythroid Cells

**Figure 3:**
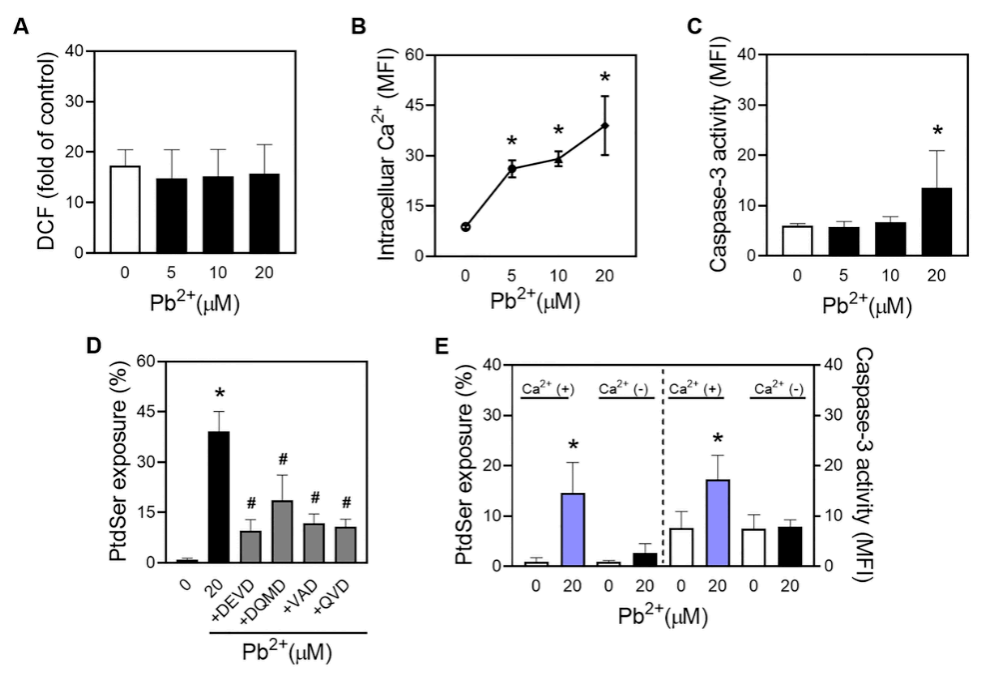
ROS-independent but [Ca^2+^]_i_-dependent eryptosis by Pb-treated erythroid cells. Isolated human erythroid cells were treated with various concentrations (0, 5, 10, and 20 μM) of Pb at 37 °C for 1 h. (**A**) DCF is a method to determine the content of intracellular reactive oxygen species by using flow cytometry. (**B**) Intracellular calcium level was examined by preloading 3 μM fluo-4 AM for 1 h. (**C**) Caspase 3 activity was measured as shown in the Materials and Methods section. (**D**,**E**) Inhibition of PtdSer exposure and caspase 3 activity was performed by preloading various caspase inhibitors or a calcium-chelating agent (TPEN) as shown in the Materials and Methods section. Values are the mean ± SD of 3–5 experiments. * Statistical difference compared with control (*p* < 0.05). ^#^ Statistical difference compared between groups (*p* < 0.05).

ROS production plays a key role in eryptosis [26]. In order to identify the function of ROS, isolated human erythroid cells were pre-treated with various concentrations (0, 5, 10, and 20 μM) of Pb at 37 °C for 1 h. No ROS production was observed in Pb-treated erythroid cells by using flow cytometry (Figure 3A). Moreover, an increase of intracellular Ca^2+^ activity ([Ca^2+^]_i_) was also regarded as one of the signals of eryptosis [22,27]. When preloading fluo-4 AM, we observed that [Ca^2+^]_i_ was significantly increased in a concentration-dependent manner after Pb treatment (Figure 3B). Caspase 3 activity is closely related to intracellular calcium level in eryptosis [28,29], and, consistently in our study, Pb treatment can successfully upregulate caspase 3 activity (Figure 3C). Indeed, both caspase 3 activation and intracellular calcium are key contributors to PtdSer externalization [30]. To further examine the role of caspase and calcium in PtdSer externalization, we preloaded various caspase inhibitors (Z-DEVD-FMK, Z-DQMD-FMK, Z-VAD-FMK, Q-VD-OPh) or TPEN as a calcium-chelating agent (Figure 3D,E-left) and found that they can successfully inhibit PtdSer externalization by Pb. Also, with TPEN, caspase 3 activity was also reversed, showing caspase 3 activity was regulated by intracellular calcium (Figure 3E, right).

### 2.4. Pb-Induced Eryptosis Participated in Procoagulant Activity and Adhesion to Endothelium Cells

**Figure 4:**
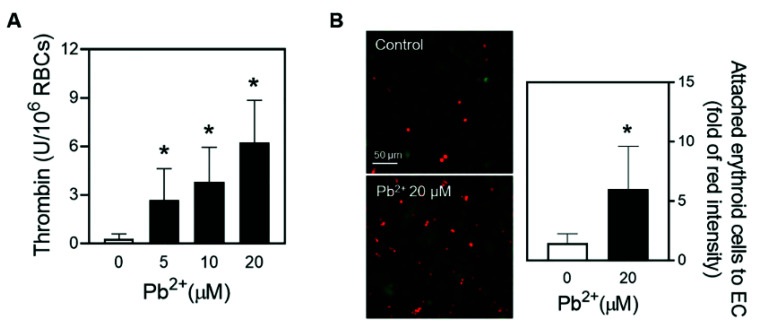
Pb-induced eryptosis participated in procoagulant activity and adhesion to endothelium cells. After isolated human erythroid cells were treated with distilled water (DW; control) or various concentrations of Pb for 1 h at 37 °C, (**A**) procoagulant activity was shown by a concentration-dependent trend of thrombin generation using prothrombinase assay (*n* = 4–7). (**B**) Enhanced adherent erythroid cells to HUVECs were detected by fluorescence microscope as described in the Materials and Methods section. Endothelial cells (green fluorescence); erythroid cells (red fluorescence) (*n* = 4). Values are the mean ± SD of 4–7 experiments. * Statistical difference compared with control (*p* < 0.05). Scale bars (**B**): 50 μm.

Many studies have shown the procoagulant activity of exposed PtdSer in erythroid cells [31,32]. With prothrombinase assay as mentioned in the Materials and Methods section, procoagulant activity of human erythroid cells was shown by a concentration-dependent trend of thrombin generation after 1 h treatment with various concentrations of Pb at 37 °C (Figure 4A). In addition, fluorescence microscopy showed that Pb treatment could also enhance adherent erythroid cells to HUVECs (Figure 4B).

### 2.5. In Vivo Evaluation of Pb-Induced Thrombosis in a Venous Thrombosis Mice Model

**Figure 5:**
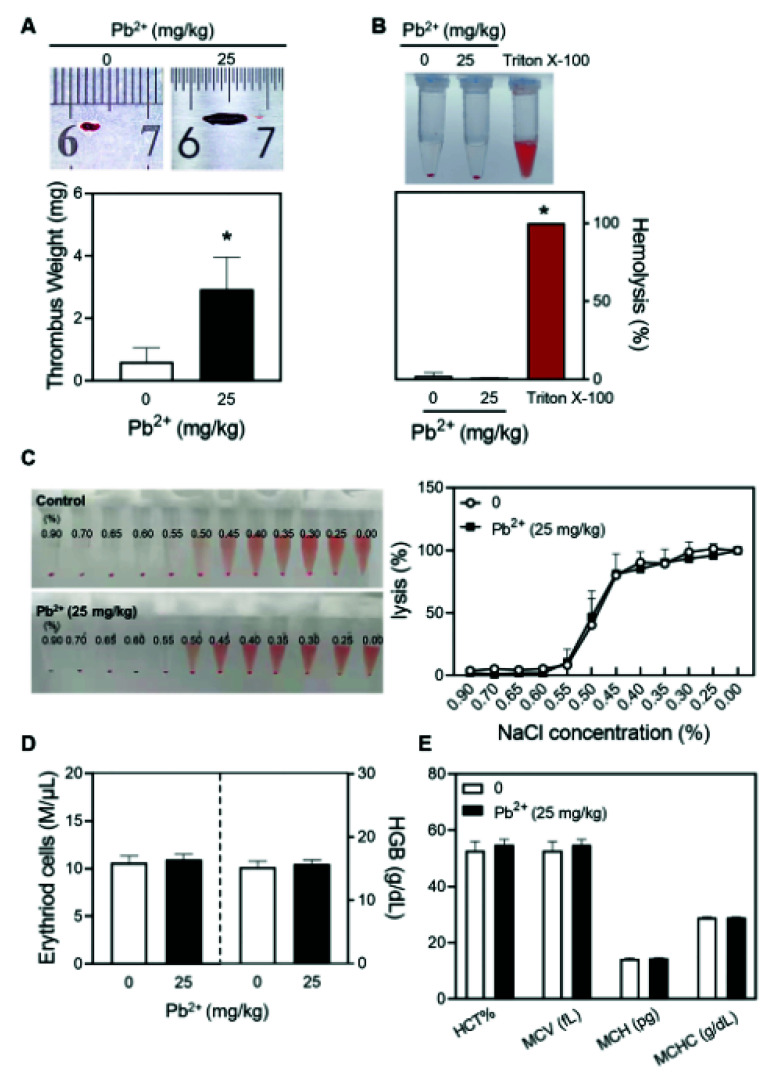
Pb can cause thrombus formation prior to hemolysis in mice. After 25 mg/kg Pb exposure to mice for 1 h was employed, (**A**) thrombus formation was observed and thrombus weight was determined. In addition, (**B**) in vivo hemolysis and (**C**) erythrocyte osmotic fragility (left, imaging data; right, percentage of lysis in different concentrations of NaCl) were performed. (**D**) The counts of erythroid cells and hemoglobin (HGB) content, (**E**) hematocrit percentage (HCT%), mean corpuscular volume (MCV), mean corpuscular hemoglobin (MCH) and mean corpuscular hemoglobin concentration (MCHC) were detected by using a hematology analyzer. Values are mean ± SD of 3–4 independent experiments, * Statistical difference compared with control (*p* < 0.05).

For the in vivo evaluation of thrombotic risks of Pb, a single dose of Pb (0 or 25 mg/kg) was intravenously injected into mice by using the venous thrombosis mice model. As a result, Pb treatment could increase thrombus weight (Figure 5A). However, no hemolysis (Figure 5B) was observed, and the osmotic fragility test showed there was no difference between control and Pb-treated mice (Figure 5C). In addition, no significant alteration was shown in the counts of erythroid cells or hemoglobin (Figure 5D) in Pb-exposed groups compared to vehicle groups. Moreover, blood from control and Pb-treated mice showed no difference in hematocrit, mean corpuscular volume (MCV), mean corpuscular hemoglobin (MCH), or mean corpuscular hemoglobin concentration (MCHC) (Figure 5E). In general, the above data indicated the thrombotic risks of Pb occurred without the occurrence of hemolysis and anemia.

Furthermore, we tested other blood cells besides erythroid cells. As a result, we found no significant alteration was involved in plasma coagulation, platelet aggregation, and thrombin generation by using human in vitro samples (Supplementary Materials, Figure S1A–C) and in vivo information (Supplementary Materials, Figure S1D–F) including platelet cell counts, platelet large cell ratio (PLCR), and platelet distribution width (PDW), mean platelet volume (MPV), as well as white blood cells.

### 2.6. Eryptosis Was Involved in Pb-Induced Thrombosis in Mice

**Figure 6:**
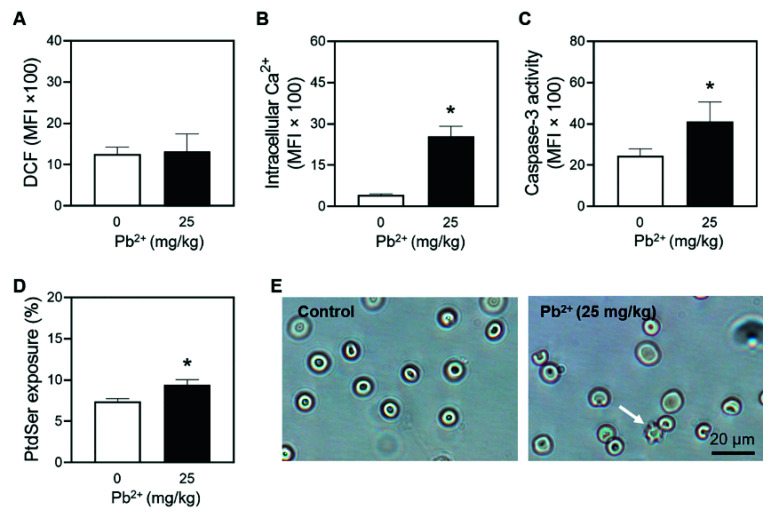
Pb-induced eryptosis in mice. After 25 mg/kg of Pb was exposed to mice for 1 h, (**A**) ROS, (**B**) intracellular calcium, (**C**) caspase 3 activity as well as (**D**) PtdSer exposure of erythroid cells in mice were all detected. In addition, (**E**) shape changes of erythroid cells were observed by using an optical microscope (white arrow: echinocytes). Values are the mean ± SD of 3–5 experiments. * Statistical difference compared with control (*p* < 0.05).

To examine whether eryptosis occurred in mice after Pb treatment, we tested ROS, intracellular calcium level, caspase 3 activity, PtdSer externalization, and shape changes here. As a result, ROS generation (Figure 6A) was not obviously increased, which was in good agreement with human in vitro results, as shown in Figure 3A. Also, [Ca^2+^]_i_ levels and caspase 3 activity in vivo showed obvious increases in Pb-treated groups (Figure 6B,C). In addition, we also observed increased PtdSer externalization (Figure 6D) and erythrocyte morphological changes (Figure 6E) in Pb-treated groups, which were consistent with our in vitro results.

## 3. Discussion

In our study, we demonstrated that Pb exposure may promote procoagulant activity of human erythroid cells and provoke thrombosis in mice prior to hemolysis in an ROS-independent but calcium-dependent eryptosis. Pb-induced procoagulant activity occurred at sub-hemolytic concentrations and thrombin generation, and adhered erythroid cells to endothelial cells were also facilitated. Here, ROS did not participate in regulating PtdSer externalization and MV generation. But interestingly, increased intracellular calcium levels can upregulate caspase 3 activity, both of which contributed to the disruption of phospholipid asymmetry and led to PtdSer externalization and MV generation, as well as shape changes into echinocytes and spherocytes. In parallel with in vitro results, in vivo relevance to thrombosis prior to hemolysis was well proven in a mice venous thrombosis model, supporting the prothrombotic risk of Pb prior to lysis.

ROS appeared to play a key role in eryptosis in previous studies [26], which seems contrary to our conclusion. However, ROS-independent events were also reported in several studies, and they demonstrated that intracellular calcium could also regulate eryptosis even without ROS production [27]. In good accordance with previous findings, we also observed that Pb-induced eryptosis was ROS-independent but calcium-dependent.

A previous study estimated that the Pb blood level of battery manufacturing workers of Western Maharashtra (Kolhapur, India), who were occupationally exposed to Pb over a long period of time (about 15 years), was found to be in the range of 25.8–78.0 μg/dL (approximately 1.2–3.8 μM), whereas that of the control group was 2.8–22.0 μg/dL (approximately 0.1–1.0 μM) [33]. This is not far from the effective level in our study (5 μM for 1 h), which can immediately induce PtdSer externalization and facilitate thrombin generation, indicating a prothrombotic risk. More seriously, if with a prolonged exposure time and repeated exposure, the toxicity level will be much lower and prothrombotic risk will come earlier than we expected, then we must pay more attention to its potential thrombotic hazards at lower exposure levels.

We observed that thrombosis occurred with Pb treatment before hemolysis, which might suggest whether anemia should be considered as a potential risk of thrombosis in clinical practice. Retrospective studies showed that anemia was diagnosed in some patients with cerebral venous thrombosis (CVT) [34], and anemia is a risk factor for CVT [35]. Clinical studies observed that patients with hemolytic anemia manifest thrombotic complications, such as venous thromboembolism and stroke [36,37]. Here, we demonstrated that Pb can not only induce thrombosis but can occur prior to hemolysis as well, suggesting that we should pay more attention to the risk of thrombosis before hemolysis and anemia.

## 4. Materials and Methods

### 4.1. Chemicals

Lead (II) acetate (Pb), CaCl_2_, glucose, ethylenediaminetetraacetic acid (EDTA), bovine serum albumin (BSA), *N*, *N*, *N*′, *N*-tetrakis (2-pyridylmethyl) ethylene diamine (TPEN), *N*-[2Hydroxyethyl] piperazine-*N*′-[2-ethanesulfonic acid] (HEPES), sodium dodecyl sulfate, glutaraldehyde solution and osmium tetroxide, and purified human thrombin, were purchased from Sigma-Aldrich Co. (St. Louis, MO, USA). Phycoerythrin-labeled monoclonal mouse anti-human CD235a (anti-glycophorin-A-PE) and fluorescein isothiocyanate (FITC)-labeled annexin V (annexin V-FITC) were purchased from BD Pharmingen (San Diego, CA, USA). Purified human prothrombin (factor II), factor Xa and factor Va were obtained from Hematologic Technologies, Inc. (Essex Junction, VT, USA), and chromogenic substrate S2238 was purchased from Chromogenix (Milano, Italy). Human umbilical vein endothelial cells (HUVECs) and the endothelial cell growth media (EGM) kit were purchased from Lonza (Basel, Switzerland). Calcein-green AM was purchased from Invitrogen (Carlsbad, CA, USA). Thromboplastin was purchased from Instrumentation Laboratory (Lexington, MA, USA). The chloromethyl derivative of 2′,7′-dichloro-dihydrofluorescein diacetate (CM-H2DCF-DA) was obtained from Life Technologies. A caspase 3 detection kit I (FITC-DEVD-FMK) and caspase 3 inhibitors (Z-DEVD-FMK, Z-DQMD-FMK, Z-VAD-FMK, Q-VD-OPh) were purchased from Calbiochem^®^ from Merck (Darmstadt, Germany).

### 4.2. Preparation of Erythroid Cells and Measurement of Hemolysis

In order to understand the harm of lead exposure to the human body and reduce the number of experimental animals, we carried out in vitro experiments with human blood. With the approval of the Ethics Committee of the Health Service Center of China Medical University, human blood was collected by using a vacutainer with acid citrate dextrose (ACD) and a 21-gauge needle (Becton Dickinson, Franklin Lakes, NJ, USA) on each experimental day. Platelet-rich plasma and buffy coat were removed after centrifugation at 200× *g* for 15 min. Erythroid cells were washed 2 times with phosphate-buffered saline (PBS: 1.06 mM KH_2_PO_4_, 154 mM NaCl and 2.96 mM Na_2_HPO_4_ at pH 7.4) and 1 time with Ringer’s solution (125 mM NaCl, 5 mM KCl, 1 mM MgSO_4_, 32 mM HEPES, 5 mM glucose, pH 7.4), and then re-suspended in Ringer’s buffer to a final concentration of 5 × 10^7^ cells/mL with 1 mM of CaCl_2_. To measure the hemolytic response, vehicle (distilled water, DW)- or Pb-treated erythroid cells were centrifuged at 10,000× *g* for 1 min and spectrophotometrically determined at 540 nm.

### 4.3. Flow Cytometric Analysis of Phosphatidylserine Exposure and Microvesicle Generation

Human erythroid cells were incubated with a vehicle or various concentrations of Pb (0, 5, 10, and 20 μM) for 1 h. Anti-glycophorin-A-PE was used as a marker for erythroid cells and annexin V-FITC was used as a marker for phosphatidylserine (PtdSer). Negative controls for annexin V binding were stained with annexin V-FITC in the presence of 2.5 mM EDTA instead of 2.5 mM CaCl_2_. CellQuest Pro software was applied to analyze data from 10,000 events collected with the flow cytometer FACS Calibur (Becton Dickinson, Franklin Lakes, NJ, USA) equipped with an argon-ion laser emitting at 488 nm. On the basis of forward-scatter characteristics, microvesicles (MV) were identified after calibration by 1 µm standard beads.

### 4.4. Microscopic Observation

Five-hundred-μL suspended erythroid cells were attached onto a four-chambered coverslip (Lab-Tek^®^ from Thermo Fisher, Rochester, NY, USA). The process of washing was done by using Ringer’s solution containing 2% BSA. After 1 h incubation with the vehicle or various concentrations of Pb, and subsequent staining with anti-glycophorin-A-PE for 30 min, shape observation was conducted by using confocal microscopy equipped with an argon laser (TCS SP8, Leica, Solms, Germany). Excitation and emission filters were respectively set at 488 nm and 550–600 nm. For SEM observation, vehicle- or Pb-treated erythroid cells were pre-fixed with 2% glutaraldehyde solution for 1 h at 4 °C and post-fixed with 1% osmium tetroxide for 30 min at room temperature in the hood. Then the samples were dehydrated serially with 50%, 70%, 80%, 90%, and 100% ethanol and were dried for coating with gold. A field emission scanning electron microscope (Merlin Compact FE-SEM, Zeiss, Oberkochen, Germany) was conducted to observe shape changes.

### 4.5. In Vitro Experiments

For ROS detection, isolated human erythroid cells were pre-incubated with 5 μM CM-H2DCF-DA for 30 min at 37 °C. Then the cells were treated with various concentrations (0, 5, 10, and 20 μM) of Pb at 37 °C for 1 h, and the fluorescence of intracellular DCF was measured by using flow cytometry. In order to detect the intracellular calcium increase ([Ca^2+^] _i_), erythroid cells were pre-loaded with 3 μM Fluo-4AM for 1 h at 37 °C in the dark and the fluorescence of Fluo-4 was analyzed in a flow cytometer. For evaluation of caspase 3 activity, after erythroid cells were treated with Pb, caspase 3 inhibitors (Z-DEVD-FMK, Z-DQMD-FMK, Z-VAD-FMK, Q-VD-OPh were conjugated to FITC as the fluorescent in situ marker) were added to 300-μL suspended erythroid cells for 30 min incubation in a thermomixer (37 °C, 1000 rpm, dark). After centrifugation (1000× *g* for 5 min) and being washed twice, flow cytometry was used to detect resuspended cells. TPEN (100 μM, 5 min), a calcium-chelating agent, was used prior to Pb exposure, and inhibition of PtdSer exposure and caspase 3 activity were obtained by using flow cytometry.

### 4.6. Prothrombinase Assay

After treatment with Pb for 1 h, suspended erythroid cells were centrifuged and washed twice with PBS. Then samples were incubated with 5 nM factor Xa and 10 nM factor Va in Tyrode buffer (134 mM NaCl, 10 mM HEPES, 5 mM glucose, 2.9 mM KCl, 1 mM MgCl_2_, 12 mM NaHCO_3_, 0.34 mM Na_2_HPO_4_, 0.3% BSA, and 2 mM CaCl_2_ at pH 7.4) for 3 min at 37 °C. After adding 2 μM prothrombin, thrombin formation was initiated. Three minutes after the addition of prothrombin, an aliquot of the suspension was transferred to a tube containing stop buffer (50 mM Tris-HCl, 120 mM NaCl, and 2 mM EDTA at pH 7.9). The chromogenic substrate S2238 was used to determine thrombin activity. The rate of thrombin formation from the change in absorbance was calculated at 405 nm by using a calibration curve generated with thrombin.

### 4.7. Fluorescence Observation of Adhered Erythroid Cells to Human Umbilical Vein Endothelial Cells (HUVECs)

Endothelial cells (2 × 10^4^ cells) were seeded into 4-well-chamber for 2 days and stained with calcein green for 20 min. Pb-treated erythroid cells were washed 1 time and resuspended in EBM-2 to a cell concentration of 5 × 10^7^ cells/mL. HUVECs were washed 2 times with EBM-2, and Pb-exposed erythroid cells were layered onto a confluent HUVEC monolayer, before HUVECs were incubated for 60 min at 37 °C. After the incubation, the chambers were flushed once with EBM-2 to remove nonadherent erythroid cells, and glycophorin A-PE was added for staining erythroid cells. Adhered erythroid cells to HUVECs were monitored by using fluorescent microscopy.

### 4.8. In Vivo Experiments

**Scheme 1:**
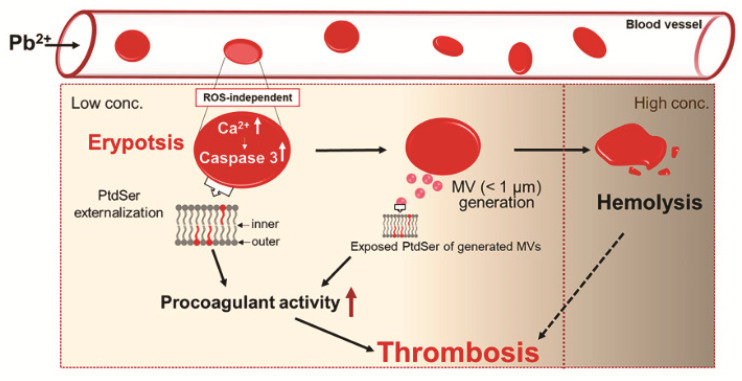
Before hemolysis, lead causes PtdSer externalization and MV generation of erythroid cells through intracellular calcium-dependent eryptosis, which leads to the increase of procoagulant activity of erythroid cells and induces thrombosis.

First, in order to reduce the number of animals used, the increased thrombus weight (Figure 5A), hemolysis (Figure 5B), and blood cell analysis (Figure 5C) in the animal experiment were all from the same batch of mice; that is, 4 mice in the control group and 3 mice in the Pb group. In addition, 1% pentobarbital sodium was used as anesthesia before an operation to reduce pain and discomfort in mice. Namely, we applied a single dose of Pb (0 or 25 mg/kg) by intravenous injection into a left femoral vein, then infused 500-fold diluted thromboplastin for 1 min to induce thrombus formation. Stasis was initiated by tightening the two blood vessels, first the proximal and the distal thereafter. The abdominal cavity was temporarily closed, and the blood vessel was maintained for 15 min. After reopening the abdomen, the ligated venous segment which was 1-cm long was excised and opened longitudinally to gain the thrombus. The isolated thrombus was blotted of excess blood, immediately weighed, and the picture of the thrombus also was observed by camera (Scheme 1).

Whole blood samples which were obtained from the above mice’s whole blood was centrifuged (10,000× *g* for 1 min) after being incubated with Pb treatment, and the extent of hemolysis was determined spectrophotometrically at 540 nm. Ringer’s solution and RBCs lysed with Triton X-100 were used as blank and 100% hemolysis, respectively.

After Pb treatment (0 or 25 mg/kg), blood was collected from mice tails (EDTA-2K as anticoagulant agent), and blood cell analysis was performed on an all-automated hematology analyzer (IDEXX ProCyte Dx).

Regarding the in vivo erythrocyte osmotic fragility test, a sodium chloride solution (pH = 7.4) with increased concentrations (0, 0.25, 0.30, 0.35, 0.40, 0.45, 0.50, 0.55, 0.60, 0.65, 0.70, and 0.90%) was prepared, and 10 μL heparinized venous blood was added to the test tube containing each solution. Each tube was gently mixed and incubated at 37 °C for 1 h. The tubes were centrifuged at 3000× *g* for 1 min. The absorbance of the supernatant of the test tube containing 1% saline was set to zero, and the absorbance of each supernatant was measured at 540 nm. lysis% = ((OD_sample_ − OD_1% NaCl_)/(OD_0%_ − OD_1% NaCl_)) × 100.

After 25 mg/kg of Pb was exposed to mice for 1 h, ROS, intracellular calcium, caspase 3 activity and PtdSer exposure of erythroid cells in mice were all detected by using flow cytometry (Refer to Section 4.3 and Section 4.5 for details). In addition, shape changes of erythroid cells were observed by using an optical microscope (Refer to Section 4.4 for details).

### 4.9. Statistical Analysis

The means and standard errors of means were calculated for all treatment groups. The data were subjected to a two-way analysis of variance followed by Duncan’s multiple range test or a Student’s *t*-test to determine which means were significantly different from the control. In all cases, a *p*-value of < 0.05 was used to determine significant differences.

## 5. Conclusions

Collectively, our study observed that Pb exposure may trigger thrombosis before hemolysis and anemia by adjusting ROS-independent but calcium-dependent eryptosis. It may provide a new idea for public health to focus on thrombosis and related diseases caused by lead exposure rather than hemolysis and anemia.

## Data Availability

The data and material that support the findings of this study are available from the corresponding author upon reasonable request. Code availability: Not applicable.

## Supplementary Materials

The following supporting information can be downloaded at: www.mdpi.com/article/10.3390/ijms23137008/s1, Figure S1: Estimation of platelets and white blood cells after Pb exposure.
