# Supplementary material for: Pb-Induced Eryptosis May Provoke Thrombosis Prior to Hemolysis
Source: Int J Mol Sci. 2022 Jun 23;23(13):7008. doi: 10.3390/ijms23137008 (PMC9266547; doi:10.3390/ijms23137008)
Supplement: Supplementary file 1 [file ijms-23-07008-s001.zip › ijms-1665885-supplementary.pdf]

Supplementary Materials:

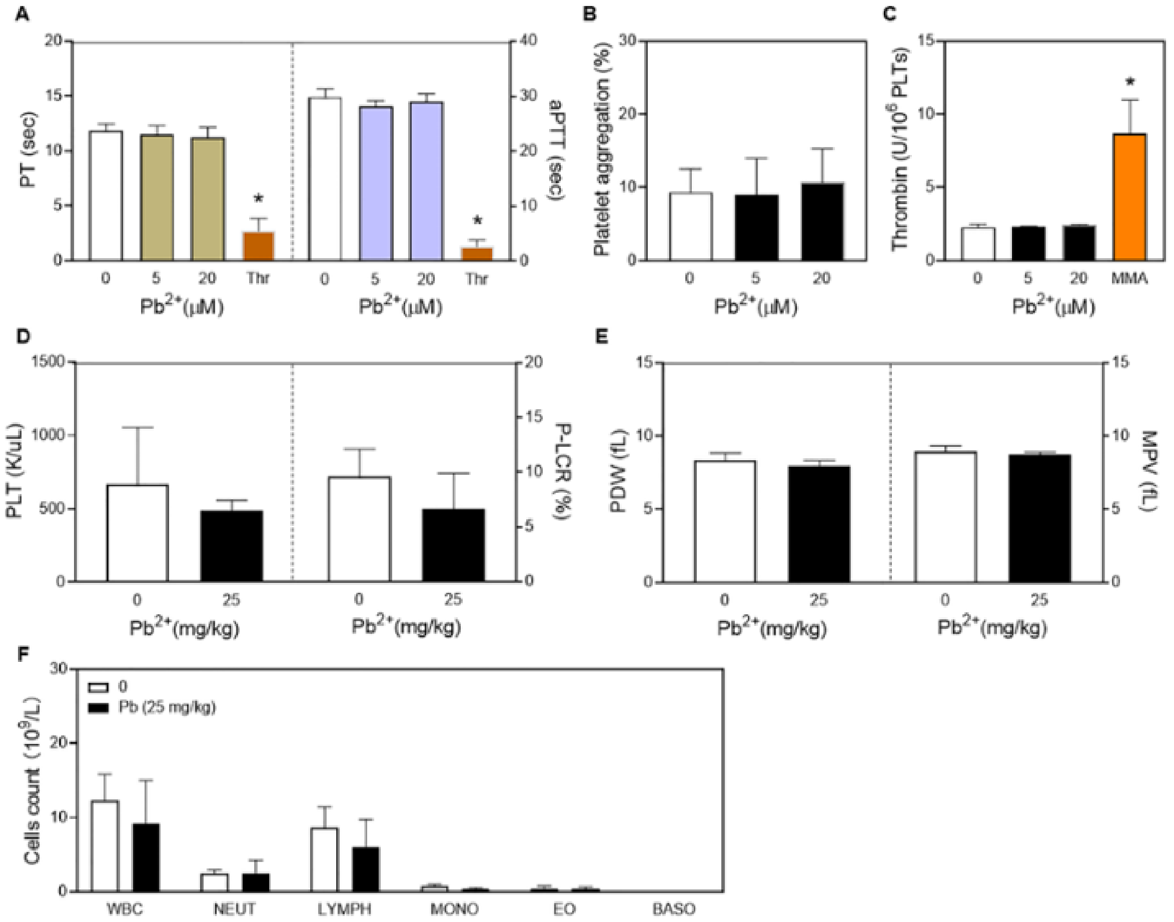

Figure S1. Estimation of platelets and white blood cells after Pb exposure. (A) Plasma coagulation was evaluated by prothrombin time (PT) and activated partial thromboplastin time (aPTT) after isolated human plasma was incubated with 0, 5 and 20 μM Pb for 1 h. Thrombin (2 U/ml) was used as positive control. **(B)** Platelet aggregation and **(C)** thrombin generation were tested using isolate human platelets after Pb treatment. Monomethylarsonous acid (MMA(III)) (50 μM) was used as positive control. **(D)** After 25 mg/kg Pb exposure to mice for 1 h, the platelets counts, P-LCR% (platelet large cell ratio, PLCR), **(E)** PDW (platelet distribution width, PDW), MPV (mean platelet volume, MPV) were all tested. In addition, **(F)** WBC (White blood cell, WBC), NEUT (Neutrophil, NEUT); LYMPH (Lymphocytes, LYMPH), MONO (Monocytes, MONO), EO (Eosinophil, EO), BASO (Basophil, BASO) were also determined using hematology analyzer. Values are mean ± SD of three independent experiments, * Statistical difference compared with control ($p < 0.05$).
